# Prosthetic Management of an Exenterated, Irradiated Orbital Defect Reconstructed with a Perforator Flap

**DOI:** 10.1055/s-0044-1801773

**Published:** 2025-01-15

**Authors:** Sandeep Gurav, Gurkaran Preet Singh, Radhika Jain, Ameya Bindu, Sagar Kulthe

**Affiliations:** 1 Dental and Prosthetic Surgery, Tata Memorial Hospital, CI of Homi Bhabha National Institute, Mumbai, Maharashtra, India; 2 Plastic and Reconstructive Surgery, Tata Memorial Hospital, CI of Homi Bhabha National Institute, Mumbai, Maharashtra, India; 3 Division of Anaplastology, Dental and Prosthetic Surgery, Tata Memorial Hospital, CI of Homi Bhabha National Institute, Mumbai, Maharashtra, India

We describe a prosthetic procedure that involves secondary flap debulking of the medial sural artery perforator (MSAP) flap covering an exenterated orbital defect and eventual placement of a cosmetically acceptable silicone-based orbital prosthesis.

**Fig. 1:**
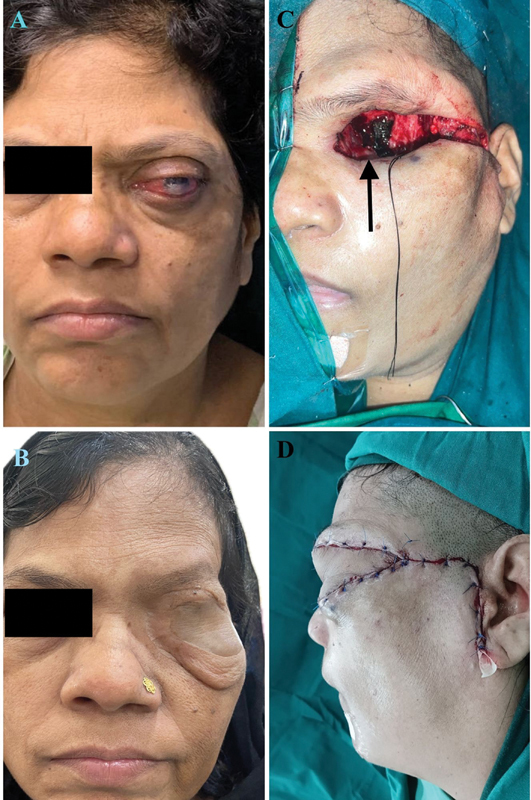
( **A** ) Preoperative image showing recurrent adenoid cystic carcinoma of the left lacrimal gland. ( **B** ) Postoperative image at 1.5 years of follow-up after surgical reconstruction. ( **C** ) Intraoperative image of the orbital surgical defect with the *arrow* pointing at the medial bony wall. ( **D** ) Reconstruction with the medial sural artery perforator flap.

A 51-year-old woman underwent orbital exenteration along with left orbital wall excision for a recurrent adenoid cystic carcinoma (grade 3) of the left lacrimal gland in July 2022 ( Fig. 1 ). The defect was primarily reconstructed with MSAP. The patient received adjuvant radiation therapy to tumor bed at a dose of 64 Gy in 32 fractions 6 weeks postsurgery. The postoperative follow-up period was uneventful.

**Fig. 2:**
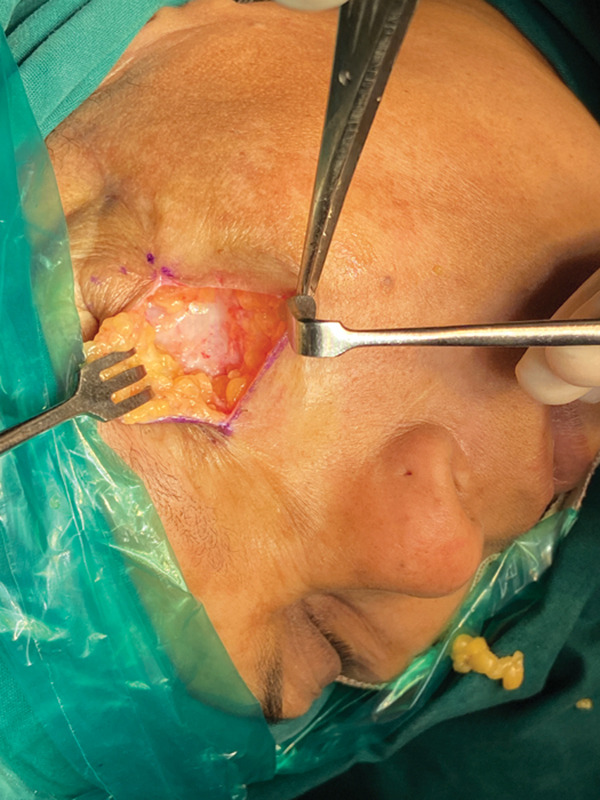
Highlight of the secondary flap thinning procedure where a midline incision was made and a subdermal full-thickness flap was raised. The adipose tissue was liberally removed, ensuring some fat is retained over the orbital bone.

She reported to our dental and prosthetic oncology unit 1 year postoperatively with a desire for an improved cosmesis. On assessment, a left exenterated defect was noted, covered with a healthy, bulky flap. The native skin was sagging at the inferior margin of the defect ( Fig. 1 ). One year had been completed postradiotherapy (PORT) and the patient was having a satisfactory Eastern Cooperative Oncology Group (ECOG-1) performance status. She was taken up for prosthetic space creation by a secondary flap thinning procedure, after corroboration with the department of plastic surgery. 1 Under general anesthesia, a midline incision was made and a subdermal full-thickness flap was raised. The adipose tissue was liberally removed, ensuring some fat was retained over the orbital bone ( Fig. 2 ). The end point of debulking was to obtain a depression similar to the depth of the contralateral normal eye. One week postoperatively, she was given a transitional acrylic conformer to ensure sustained gentle pressure on the healing tissues that served as a scaffold.

**Fig. 3:**
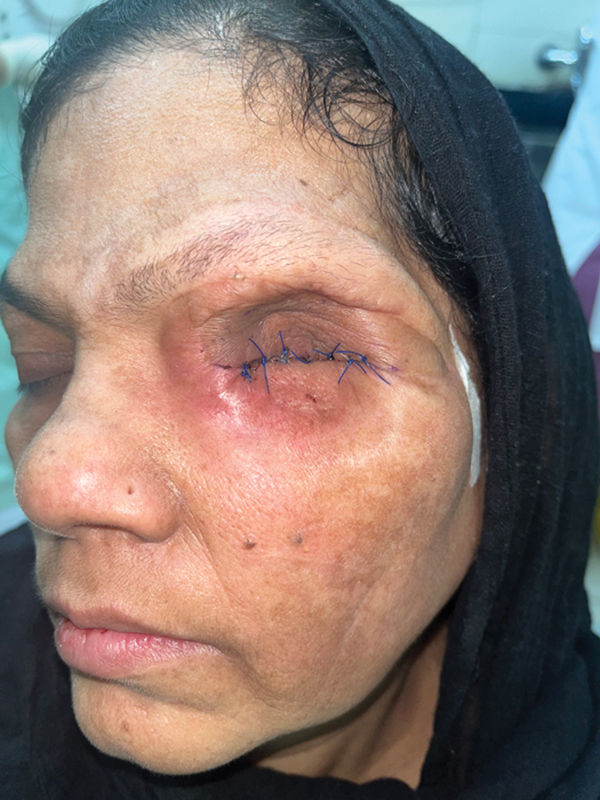
The final well-settled, healed orbital cavity ready for placement of the final adhesive-retained silicone orbital prosthesis.

**Fig. 4:**
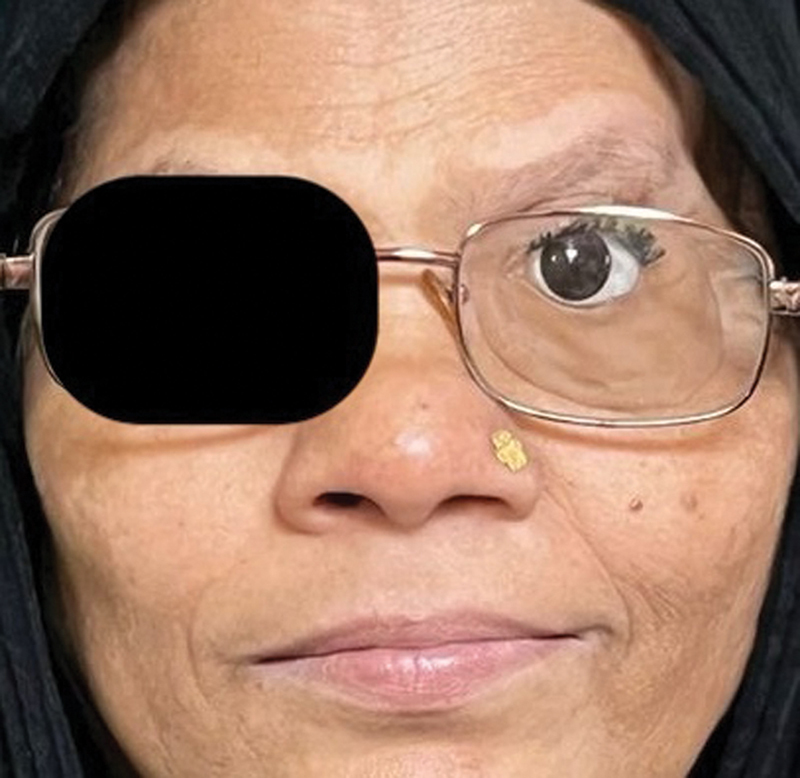
Final adhesive-retained silicone prosthesis with satisfactory aesthetics and retention.

One month later, when the final contours were achieved ( Fig. 3 ), we started with the fabrication of the final silicone orbital prosthesis. The prosthesis was processed from room temperature vulcanizing silicone (M511, Technovent) by our team of anaplastologists. Clinically, it was evaluated for fit, aesthetics, and comfort. The main mode of retention of the prosthesis was adhesive based as there were no anatomical undercuts to harness ( Fig. 4 ). Also, the fact that frontal bones were radiated precluded the possibility of orbital endosteal implants to aid in retention of the prosthesis.

MSAP flap is a versatile fasciocutaneous flap that is used for reconstruction of small to medium-sized head and neck defects. 2 As there was no muscle component in the MSAP flap, thickness of the resultant debulked tissue proved ideal for the prosthetic space creation. 3 We believe that careful planning regarding the appropriate time to intervene for various procedural steps such as time of debulking (1 year after PORT), time to place the conformer (1 week after initial healing), and duration of placement of conformer (at least 4 weeks) ensured sufficient time for healing and achieving satisfactory results. The fragile, irradiated tissue bed was cautiously manipulated by applying only gentle pressure with the use soft-tissue reline material under the acrylic conformer.

We could successfully demonstrate that it is possible to rehabilitate complex orbital defects by careful execution of soft-tissue procedures and use of innovative prosthetic techniques. The patient has been using her prosthesis regularly and attends her social and religious meetings with bolstered confidence.
